# Biological Soil Crusts as Ecosystem Engineers in Antarctic Ecosystem

**DOI:** 10.3389/fmicb.2022.755014

**Published:** 2022-03-22

**Authors:** Andrea Barrera, Ian S. Acuña-Rodríguez, Gabriel I. Ballesteros, Cristian Atala, Marco A. Molina-Montenegro

**Affiliations:** ^1^Instituto de Ciencias Biológicas, Universidad de Talca, Talca, Chile; ^2^Instituto de Investigación Interdisciplinaria (I3), Universidad de Talca, Talca, Chile; ^3^Facultad de Ciencias, Instituto de Biología, Pontificia Universidad Católica de Valparaíso, Valparaíso, Chile; ^4^Facultad de Ciencias del Mar, Centro de Estudios Avanzados en Zonas Áridas (CEAZA), Universidad Católica del Norte, Coquimbo, Chile; ^5^Centro de Investigación en Estudios Avanzados del Maule (CIEAM), Universidad Católica del Maule, Talca, Chile

**Keywords:** soil biocrusts, *Colobanthus quitensis*, enzymatic process, metabarcoding, Antarctic ecosystem

## Abstract

Biological soil crusts (BSC) are considered as pivotal ecological elements among different ecosystems of the world. The effects of these BSC at the micro-site scale have been related to the development of diverse plant species that, otherwise, might be strongly limited by the harsh abiotic conditions found in environments with low water availability. Here, we describe for the first time the bacterial composition of BSCs found in the proximities of Admiralty Bay (Maritime Antarctica) through 16S metabarcoding. In addition, we evaluated their effect on soils (nutrient levels, enzymatic activity, and water retention), and on the fitness and performance of *Colobanthus quitensis*, one of the two native Antarctic vascular plants. This was achieved by comparing the photochemical performance, foliar nutrient, biomass, and reproductive investment between *C. quitensis* plants growing with or without the influence of BSC. Our results revealed a high diversity of prokaryotes present in these soil communities, although we found differences in terms of their abundances. We also found that the presence of BSCs is linked to a significant increase in soils’ water retention, nutrient levels, and enzymatic activity when comparing with control soils (without BSCs). In the case of *C. quitensis*, we found that measured ecophysiological performance parameters were significantly higher on plants growing in association with BSCs. Taken together, our results suggest that BSCs in Antarctic soils are playing a key role in various biochemical processes involved in soil development, while also having a positive effect on the accompanying vascular flora. Therefore, BSCs would be effectively acting as ecosystem engineers for the terrestrial Antarctic ecosystem.

## Introduction

Understanding the presence of microbial communities in soils and their potential role and impact on vascular plants could be a key aspect to understanding the dynamics of ecosystems, especially in the most inhospitable environments. In recent years, one of the most studied biological soil communities, mainly due to its high coverage worldwide and its positive effect on its surrounding environment, has been Biological Soil Crusts (BSCs) (Belnap, 1993; Cantón et al., 2021). These communities strongly adapted to extreme conditions such as prolonged dry conditions and extremely high and low temperatures (Kauffman and Pyke, 2001) cover approximately 12% of the world’s land surface (Maier et al., 2018; Rodriguez-Caballero et al., 2018). These BSCs exist in almost all ecosystems where vascular plants have low soil cover (Maestre et al., 2017). Biological Soil Crusts are formed by consortiums of different organisms, cyanobacteria, algae, lichens, and some bryophytes being considered as the most notorious, among other heterotrophic bacteria, archaea, and fungi (Maestre et al., 2011). This soil community forms a complex amalgamation of soil particles and organisms in the top millimeters of soil (Chamizo et al., 2017; Williams et al., 2017).

These complex communities may act as modulators of soil resource availability (such as water and nutrients), which in turn may favor the establishment of other organisms, like vascular plants (Cantón et al., 2020). Therefore, BSCs have been considered as ecosystem engineers in some harsh environments (*sensu* Jones et al., 1994), as they, directly and indirectly, regulate resource availability, while influencing other species, due to the physical and chemical changes they induce on the surrounding soil (Jones et al., 1997; Bowker et al., 2013). Biological Soil Crusts can aggregate and stabilize the soil (by reducing soil erosion), while regulating critical soil functions, such as moisture retention and carbon and nitrogen fixation (Belnap et al., 2001; Aranibar et al., 2022). This BSC-mediated increase in nutrients and water availability positively affect local plant species (Song et al., 2017). Indeed, since BSCs are pioneers in the colonization of inert substrates, many studies highlight their role in ecological succession, by promoting initial soil formation and allowing the subsequent establishment of vascular plants (DeFalco et al., 2001; Weber et al., 2016; Concostrina-Zubiri et al., 2019).

Biological Soil Crusts (BSCs) also participate in the incorporation of nutrients into the soil, such as P (Baumann et al., 2017), and increase the concentration of other essential elements such as K, Fe, Cl, Mn, and S (Lange et al., 1994; Rogers and Burns, 1994; Belnap and Lange, 2001; Harper and Belnap, 2001; Bowker et al., 2016). On the other hand, these microbial communities also promote the presence of secondary metabolites and amino acids in the soil (Swenson et al., 2018) by enhancing enzymatic activity and nutrient cycling processes (Benvenutto-Vargas and Ochoa-Hueso, 2020). This, in turn, can positively impact vascular plants’ germination, growth, nutritional state, physiological performance, distribution, and abundance (Belnap et al., 2001; Shepherd et al., 2002; Zhang and Nie, 2011; Zhang and Belnap, 2015), especially in resource-limited environments.

Based on the classic definition of biological soil crusts (biocrust) by Belnap et al. (2001), BSCs are considered as the “living skin” at the soil surface in many low productivity ecosystems around the world, including water- and cold-limited environments and early successional sites. Thus, Antarctica becomes an interesting search and characterization model of these biological communities, mainly because characteristics of the environments where they have been described are present, such as soils with low water availability, low nutrient content, low vascular plants cover, and adverse environmental conditions. Several studies have been carried out in the Antarctic, showing the positive effects of thin layers of vascular plants, mosses, or lichens on the physicochemical properties of the soil and the microbial communities (Hrbácek et al., 2020; Prater et al., 2021; Ball et al., 2022), and few studies have focused on the BSCs of Antarctica, most of them being centered on continental Antarctica (Green and Broady, 2001; Büdel and Colesie, 2014; Colesie et al., 2014).

In the case of Antarctic terrestrial ecosystems, two native vascular plants, *Deschampsia antarctica* and *Colobanthus quitensis*, coexist as dominant elements, along with a mosaic of different BSCs (Molina-Montenegro and Cavieres, 2010; Parnikoza et al., 2011). While both plants grow successfully in the Antarctic tundra, their ecological strategies vary greatly; *D. antarctica* has several physiological traits that allow it to survive the extreme conditions of Antarctica (Sáez et al., 2019), but *C. quitensis* seems to strongly depend on mutualistic interaction with microorganisms (Torres-Díaz et al., 2016; Gallardo-Cerda et al., 2018; Acuña-Rodríguez et al., 2020). Thus, we believe that it is highly important to describe the composition of Maritime Antarctic BSCs (King George Island, South Shetland Islands) and their effect on the physical–chemical properties of the soil, and to evaluate their effect on the ecophysiological performance of *Colobanthus quitensis*. To achieve this, we used a metabarcoding approach to characterize the composition and diversity of the most conspicuous biocrust-like microbial soil patches of King George Island in maritime Antarctica, and we evaluated their effect on soil properties such as water retention, enzymatic activity, and nutrient content. In addition, by means of a controlled manipulative experiment, we quantified the contribution of BSCs on the performance and development of *C. quitensis* by measuring leaf nutrient content, biomass gain, photochemical efficiency (Fv/Fm), and reproductive output (number of flowers) among individuals growing with and without the influence of BSCs. Taken together, our experimental setup allowed us to determine whether BSCs had a positive effect on both soils and plants, and how much variation in terms of prokaryotic organisms is found among different BSCs from the same environment.

## Materials and Methods

### Study Site and Biological Samples

**FIGURE 1:**
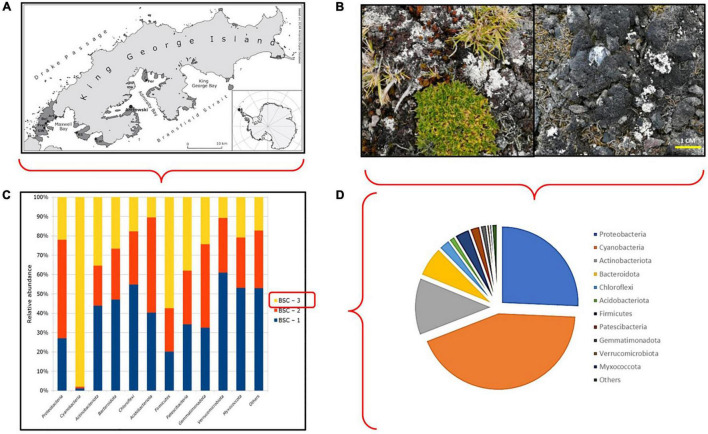
**(A)** Study site. Map of the study area in Thomas Point, close to Henryk Arctowski Polish base, King George Island (62°09′S; 50°28′W). **(B)** Hill close to Henryk Arctowski base showing the interaction between biological crusts and vascular plant communities at the study site and biological crust types included in this study. **(C)** Amplicon metabarcoding information. Phylum relative abundance in all BSC samples, according to 16S sequence. **(D)** Amplicon metabarcoding information. Phylum relative abundance in BSC-3 sample, according to 16S sequence.

All samples (biocrusts and plants) were collected near “Henryk Arctowski” (Figure 1A) Polish Station in Admiralty Bay, King George Island (62°09′S; 50°28′W). This area is characterized by a mean annual temperature of −2.8°C and a mean rainfall of 700 mm, mostly as snow during the winter season or rain during the summer season (Kejna et al., 2013). Biocrusts were sampled on three different sites (site 1: 62°9′47.11″S, 58°27′32.07″W; site 2: 62°9′51.79″S, 58°27′46.18″W; site 3: 62°9′33.24″S, 58°28′14.66″W). Sampling sites were selected based on the observation of *C. quitensis* plants growing in apparent association with BSCs to maximize BSC diversity naturally co-occurring with plants in maritime Antarctica (Figure 1B). Seven samples of biological soil crusts were obtained from three different sites. Each of these seven samples was pooled into a single sample per site (BSC-1, BSC-2, and BSC-3, respectively). To avoid crust surface break-up during sampling, crusts were moistened (Song et al., 2017) and a 9-cm Petri dish was gently pushed over the substrate, as described by Jung et al. (2018). After sampling, the obtained BSC disks (about 5 mm thick) were air dried at room temperature for 2–3 days. Then, Petri dishes containing dried BSC samples were sealed with parafilm (GmbH, Wertheim, Germany), transported, and stored at Universidad de Talca at 4°C for further analysis. In addition, 21 bare soil samples (with no apparent organic cover) were collected with a sterile shovel, 1 m away from a BSC collection point, and stored in sterile 50-ml screwcap tubes. *Colobanthus quitensis* plants were sampled as part of the 56th Antarctic Scientific Expedition (ECA-56) of the Instituto Antártico Chileno (INACH) during the growing season of 2019–2020. Thirty individuals were collected, along with soil attached to the roots, and were put in a plastic box (120 × 70 × 50 cm). All plants were well watered until their arrival 1 week after to the Instituto de Ciencias Biológicas at Universidad de Talca (Chile).

### Biological Soil Crust Characterization

Biological Soil Crust (BSC) prokaryotic characterization was conducted using the methodology described by Abed et al. (2019). For each sampling site, samples were pooled before metabarcoding analysis. Therefore, three different pools of biocrust samples were obtained from different sites. For each pool, total DNA was extracted in a laminar flow cabinet to prevent environmental contamination from 0.25 g of crust biomass using the PowerSoil DNA kit (MoBio Laboratories Inc., Carlsbad, United States) following manufacturer instructions. DNA sample quality was assessed using 1% agarose gel electrophoresis, while DNA concentration and purity were estimated by spectrophotometry (Nanodrop Technologies, Wilmington, United States) at 260 nm and OD260/280 ratio > 1.8. All DNA samples were sent to MACROGEN (Seoul, South Korea) to produce amplicon libraries for the V4 hypervariable region of the 16S rRNA gene, by using 16S amplicon library preparation protocol and sequencing (Illumina, San Diego, United States). Briefly, an amplicon library was constructed by PCR amplification using single-indexed primers flanked by Illumina standard adapter sequences. According to this methodology, two PCR steps were necessary; the first step was for the identification of bacteria, the targeted gene region was amplified from the crust DNA samples using the Illumina overhang adapter sequences attached to locus-specific primers S-D-Bact-0341-b-S-17 (forward primer, 5′-CCTACGGGNGGCWGCAG-3′) and S-D-Bact-0785-a-A-21 (reverse primer, 5′-GACTACHVGGGTATCTAATCC-3′). These are considered the most suitable primer pairs to conduct PCR-based soil and plant-associated bacterial microbiome diversity studies (Klindworth et al., 2013; Thijs et al., 2017). Then, a second amplification step attached unique indexing primers to the PCR product to identify multiplexed samples after sequencing steps (Kraler et al., 2016). Finally, libraries were sequenced on an Illumina MiSeq (Paired End, 2 × 300bp) at Macrogen Inc. (Seoul, Korea).

### Bioinformatics Analysis

16S Illumina metabarcoding libraries (*N* = 3) were analyzed using the open-source software pipeline Quantitative Insights Into Microbial Ecology package (QIIME2, 2021.4^1^) (Bolyen et al., 2019). This pipeline performed quality filtering and primer removal of Illumina amplicon sequencing data using DADA2 (Callahan et al., 2016). Then, QIIME2 assigned Illumina short reads into amplicon sequence variant (ASV) tables. The overall adequacy of reads generated by each sample was estimated through a rarefaction curve using QIIME2 at a depth of 20,000 reads. This ASV table was used as a reference to conduct taxonomic assignments using the Silva 138 99% 16S rRNA gene reference database classifier for prokaryotes.^2^ Finally, the number of reads assigned to each taxon for bacterial BSCs was pooled (by adding) among samples. This approach allowed us to explore, from a global perspective, the taxonomic composition of BSC microbial communities (Simões-Silva et al., 2020).

### Physical and Chemical Effect of the Biological Soil Crust on the Soil

Due to variability in terms of BSCs microorganism composition, the following analyses were conducted using a BSC collected from site 3 (BSC-3) (Figure 1C), as it showed a higher relative proportion of Cyanobacteria compared with BSC-1 and BSC-2. Hence, to evaluate the effect of BSC-3 crust samples on soil properties, we prepared twenty 250-ml pots (16 × 13 cm), which were filled with a mixture of sterile sand, native soil, and peat in a 4:4:1 proportion, that has been used on previous studies involving Antarctic vascular plants (Barrera et al., 2020). In half of the pots (*N* = 10), we randomly placed four 1-cm^2^ BSC-3 fragments per pot soil surface, equidistantly from each other, in a cross-shaped fashion, to reduce any potential bias caused by fragmentation and/or sample distribution. As a control condition, in the other 10 pots, we used the same soil mixture but without BSC fragments (bare soil, BS). All pots were maintained in a growth chamber under controlled conditions, at a photosynthetic photon flux density (PPFD) of 350 μmol photons m^–2^s^–1^ with a photoperiod of 20/4 h light/dark, 75% relative humidity, and at 4°C. All pots were watered with 20 ml of water once per week, mimicking current water availability in Maritime Antarctica during a typical growing season (Barrera et al., 2020; Hereme et al., 2020).

Pots were kept for 90 days in the growth chambers. During that time, we evaluated the role of BSCs on physical–chemical properties of the soil by measuring soil moisture and the activity of three enzymes (β-glucosidase, urease, and dehydrogenase). Soil moisture is related to C and N biogeochemical cycles, and enzyme activity to the abundance of microorganisms (Sinsabaugh et al., 2009). Soil moisture was measured by following the methodology described in Benavent-González et al. (2018). Seven days after the first watering, we randomly selected pots with or without BSC (*N* = 3 per condition) and took 20 g of soil samples, which were weighed on a digital scale (Boeco BBL-52; 0.01 g precision). Then, each sample was dried on a stove at 110°C for 24 h, and weighed again, to assess the available water content.

Enzymatic activity was measured at 0, 30, 60, and 90 days, at 25°C and following the method proposed by Bell et al. (2014). β-Glucosidase activity was measured using 0.5 g of soil (*N* = 3 per treatment) adding 0.5 ml of 4-nitrophenyl-β-D-glucopyranoside 50 mM (PNG) as an enzymatic substrate. Results were expressed as micrograms of *p*-nitrophenol (PNP) per gram of PNG produced per hour (g PNG g^–1^ PNP h^–1^). For urease activity, we used 1 g of soil in a 0.64% v/v solution of urea to determine the amount of NH_4_^+^ produced. This was done through colorimetric methods, measuring absorbance with a spectrophotometer at 525 nm. Results were expressed as micrograms of N-NH_4_ per gram per hour. Dehydrogenase activity was determined also using 1 g of soil per pot. To each sample, we added 0.2 ml of a 0.4% v/v solution of 2(*p*-iodophenyl)-3-(*p*-nitrophenyl)-5-phenyl tetrazolium chloride (INT) as substrate. Results were expressed as micrograms of reduced iodonitrotetrazolium formazan per hour (INTF g^–1^h^–1^). All measurements considered three technical replicates and a negative control (blank, with no BSC). Finally, soil nutrient levels (N, P, K, Ca, Mg, and Na) were quantified in three 10-g samples per condition (BSC/BS). Analyses were conducted at 0, 30, 60, and 90 days. Nutrient measurements were done in the Centro Tecnológico de Suelos y Cultivos at Universidad de Talca, Talca, Chile, as described by Song et al. (2017).

### Effects of the Biological Soil Crusts on the Performance of *Colobanthus quitensis*

To evaluate the effect of BSC on the ecophysiological performance of *C. quitensis*, we conducted a manipulative experiment using 100 individuals, which were obtained through vegetative propagation in the laboratory from 30 field-collected plants using the methodology described in Zuñiga et al. (2009). All plants were maintained in 50-ml pots containing a 4:4:1 mixture of sterile sand, native soil, and peat, respectively, and were kept well-watered until they reached ∼2 cm in diameter. Then, these plants were randomly transplanted to the 20 pots with or without BSC, whose soil properties were previously characterized (5 plants/pot). Plants were transplanted equidistantly from each other and maintained in a growth chamber with the same environmental conditions described earlier. After 60 days, we measured the maximal photochemical efficiency of the PSII (Fv/Fm), leaf nutrient content, biomass, and flower number per plant.

Fv/Fm has been described as a good proxy for the overall physiological status of the plant (Molina-Montenegro et al., 2012a,b). This parameter was measured in 10 randomly selected *C. quitensis* individuals (one per pot) in each experimental condition (with or without BSC) using a portable fluorimeter (Hansatech FMS 2; Hansatech Instruments Ltd, Norfolk, United Kingdom). Prior to each measurement, the target leaf was kept in the dark for 30 min, ensuring that the data correspond to the maximal PSII efficiency (Torres-Díaz et al., 2016). Leaf nutrient content was determined in three samples (three individuals per sample) to reach the minimum weight required for this analysis. Thus, we obtained N, K, P, Ca, Mg, Mn, Zn, Cu, Fe, and B content at 0, 30, and 60 days after the beginning of the experiment. Nutrient analysis was done at the Centro Tecnológico de Suelos y Cultivos at Universidad de Talca (Chile), based on the method proposed by Song et al. (2017). To estimate the effect of the BSCs on the plant foliar biomass, we extracted and weighed at the end of the experiment the aerial part of all *C. quitensis* individuals. Fresh biomass measurements were made using a digital scale (Boeco BBL-52; precision 0.01 g). Finally, plant fitness was measured after 60 days, as the flower number per plant, in 25 individuals per experimental group (with or without BSC), which were randomly selected at the beginning of the experiment.

### Statistical Analysis

Since data were not normally distributed (enzymatic activities, nutrient concentrations), we used the Wilcoxon rank sum test (also known as Mann–Whitney *U* test) to assess the effect of the BSCs on the physical–chemical characteristics of the soil. On each time point, the nine monitored variables—soil water content, three enzymatic activities (β-glucosidase, urease, and dehydrogenase), and six nutrient concentrations (N, P, K, Ca, Mg, Na)—were tested independently between groups. Similarly, for each of the four ecophysiological traits of *C. quitensis* (i.e., Fv/Fm, and the foliar concentrations of N, P, and K), the effect of the BSCs was also analyzed with the same non-parametric method. In addition, after verifying the parametric assumptions of normality and homoscedasticity, the overall effect of the BSC on the mean fitness response of *C. quitensis* in terms of flower production and foliar fresh biomass was estimated by independent Student tests (*t*-tests) for each variable, comparing plants grown with and without the influence of BSC. All analyses were performed on the R environment and language for statistical computing v4.0.2 (R-Core Team, 2020).

### Data Repository

All sequence data raw libraries were submitted to NCBI Sequence Read Archive (SRA) and are available under the Bioproject accession number PRJNA765698. Representative ASV sequences and ASV feature tables for both 16S sequencing were deposited at Figshare platform (available at doi: 10.6084/m9.figshare.1668081).

## Results

### Biological Soil Crust Characterization

High-throughput sequencing of 16S rRNA gene amplicons from pooled samples generated 2,042,990 reads in total, representing more than 600 megabases. After DADA2 analysis, all reads were clustered into 641 ASVs; furthermore, for all samples, rarefaction curves reached a stable asymptote, suggesting the availability of sufficient reads for identification of all the bacteria present in these samples. Regarding the taxonomic analysis, 20 different bacterial phyla were found among all sites (BSC-1, BSC-2, BSC-3). Overall, the most abundant phyla were Proteobacteria (42.3%), Cyanobacteria (16.09%), Actinobacteria (12.46%), Bacterioidetes (8.76%), and Chloroflexi (4.88%). Our results show that the same phyla were found among all BSCs, but with different relative abundances (Figure 1C). As we observed a higher relative abundance of Cyanobacteria in BSC-3, we selected this sample for further manipulative studies (see Materials and methods section). In this particular sample, a dominance of Cyanobacteria (43.44%) followed by Proteobacteria (25.69%), Actinobacteria (12.12%), and Bacterioidetes (6.41%) was observed (Figures 1C,D). Finally, a total of 252 taxa (either species/genus/family/phyla levels) were found among all analyzed samples, but only 41 were shared between BSCs, while 52, 40, and 63 taxa were found exclusively in BSC-1, BSC-2, and BSC-3, respectively.

### Physical–Chemical Effect of the Biological Soil Crusts on the Soil

**FIGURE 2:**
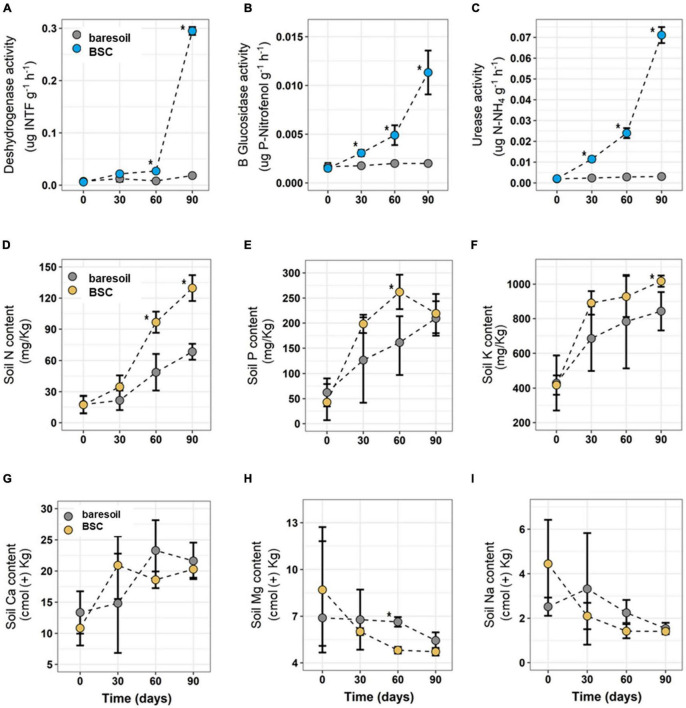
Dehydrogenase **(A)**, β-glucosidase **(B)**, and urease **(C)** enzymatic activity in soils with the presence of BSC (light-blue circles) and without BSC (BS soils, gray circles) at different times. N **(D)**, P **(E)**, K **(F)**, Ca **(G)**, Mg **(H)**, and Na **(I)** content in soils with (orange circles) or without (BS, gray circles) BSC at different times are also shown. Statistically significant differences (Wilcoxon paired test) are indicated with an asterisk. Values in the figures correspond to average ± SD.

Soil moisture, measured at the end of each watering cycle (every 7 days), was higher in soils with BSCs (26%) compared with soils without BSC (with BS, 13%). Similarly, we observed differences in enzymatic activity (for all tested enzymes) between treatments, with higher activity in BSC soils compared with BS, particularly after 90 days (Figures 2A–C). Dehydrogenase activity, associated with abundance and metabolic activity of microorganisms, increased significantly after 60 days in BSC soils, peaking at 0.30 μg INTF g^–1^ h^–1^ after 90 days from the beginning of the experiment. In contrast, BS soils reached only 0.02 μg INTF g^–1^ h^–1^ after 90 days. For the β-glucosidase activity, there were statistically significant differences between BSC and BS soils after 30, 60, and 90 days. Moreover, for BSC this enzymatic activity was an order of magnitude greater than BS at the end of the experiment (BSC = 11.30 μg PNP g^–1^ h^–1^; BS = 1.99 μg PNP g^–1^ h^–1^). Finally, urease activity (related to NH_4_^+^ availability in the soil) was higher in BSC compared with BS after 30 days. These differences also increased after 60 and 90 days from the beginning of the experiment, the time at which measured enzymatic activity was 71 μg N-NH_4_ g^–1^ h^–1^ in BSC and 3.08 μg N-NH_4_ g^–1^h^–1^ in BS, respectively.

Soil nutrient content was higher in BSC soils compared with BS, but only for N and K (Figures 2D,E). N increased significantly in BSC since day 30 compared with BS, reaching 130 mg/kg at the end of the experiment (day 90), while BS only reached 68 mg/kg in the same time (Figure 2D). For K, differences between treatments (BSC and BS) were evident only after 90 days, the time at which BSC soils reached 1,017 mg/kg *versus* only 843 mg/kg on BS (Figure 2E). No differences in P, Ca, Mg, or Mn content were found between treatments, except for a P burst after 60 days in BSC (Figures 2F–I).

### Effects of the Biological Soil Crusts on the Performance of *Colobanthus quitensis*

**FIGURE 3:**
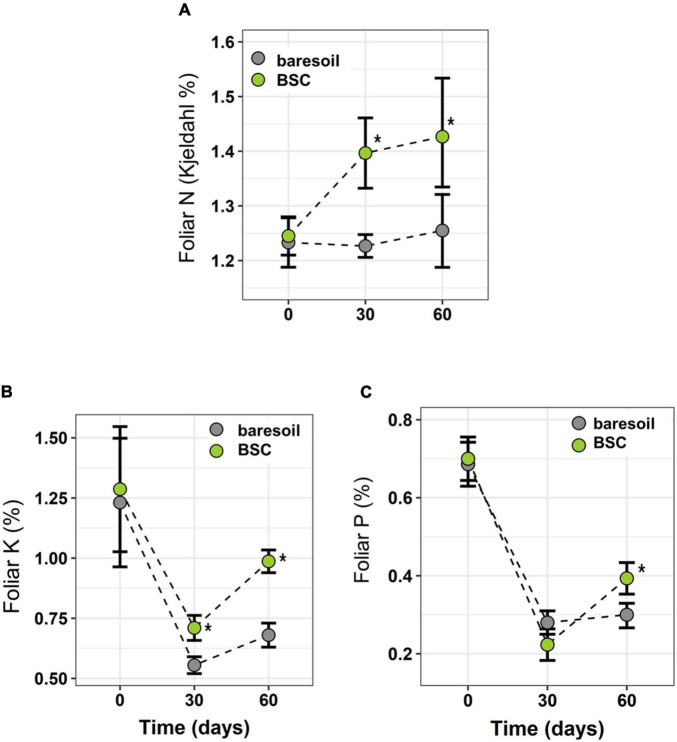
Leaf N **(A)**, K **(B)**, and P **(C)** in *C. quitensis* plants growing with (green circles) or without (BS, gray circles) BSC at different times. Box plots indicate average ± SD. Statistically significant differences (Wilcoxon paired test) are indicated with an asterisk.

**FIGURE 4:**
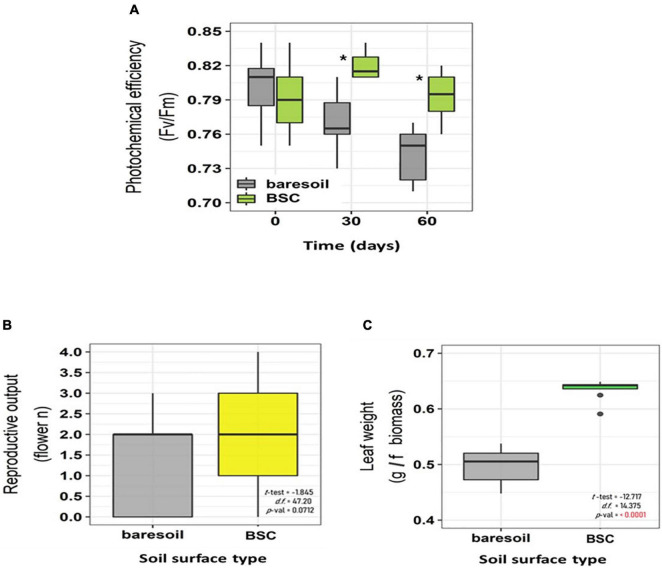
Maximal photochemical efficiency of PSII (Fv/Fm) in *C. quitensis* plants growing with (green boxes) or without (BS, gray boxes) BSC at different times **(A)**, flower number **(B)**, and fresh leaf biomass **(C)**. The values shown correspond to average ± SD. The sample size is 10 for Fv/Fm and fresh leaf biomass, and 25 for flower number. Statistically significant differences (*t*-test, *p* < 0.05) are indicated with an asterisk.

In *C. quitensis* grown with BSCs, leaf N, P, and K was higher toward the end of the experiment (day 60, Figures 3A–C) compared with individuals grown in BS. In the case of N, we observed a notorious increase in BSC plants after 30 days (Figure 3A). In terms of physiological performance, plants growing in association with BSCs had a higher Fv/Fm compared with BS plants after 60 and 90 days from the beginning of the experiment (Figure 4A). Regarding the number of flowers, we did not find statistical differences between treatments (Figure 4B). However, BSC plants had a slightly higher, although not significant, flower number than BS (55 *vs.* 33, respectively). In the case of fitness-related traits, we observed a significant increase for *C. quitensis* plants growing in presence of BSC. Plants with BSC have a higher final biomass (0.98 g) compared with plants grown in bare soil (0.77 g), while leaf biomass was also higher in BSC compared with BS (Figure 4C).

## Discussion

Our results suggest that the Antarctic soil microbial communities evaluated in this study act as a true biological crust, with a positive influence on soil properties and on vascular plants growing in their vicinity. Specifically, this positive effect could be explained by the role of BSCs roles in the increase in water availability and nutrients in the soil. Therefore, BSCs communities could also be considered ecosystem engineers in land surfaces found in Maritime Antarctica, similar to what has been described for other environments, such as dry deserts (García-Moya and McKell, 1970; Barger et al., 2016; Delgado-Baquerizo et al., 2016; Ferrenberg et al., 2018). In addition, the metabarcoding analysis showed that the predominant prokaryote phyla were Cyanobacteria, Proteobacteria, and Actinobacteria, similar to those found in other BSC studies (Abed et al., 2019) and in agreement with previous reports on Antarctic BSCs, which have shown a high proportion of Cyanobacteria, mostly from the Nostocales and Oscillatoriales (Büdel and Colesie, 2014; Ferrenberg and Reed, 2017; Williams et al., 2017; Darrouzet-Nardi et al., 2018).

The increase in water availability related to BSCs suggests that these microbial communities act as biological water-retention structures and, through their effect on water content, impact different biotic interactions and the plant community structure in dry environments (Miranda et al., 2011; Luzuriaga et al., 2012). In addition, the increase in soil moisture would also increase the duration of humidity and available water content, which would also have a positive impact on both biocrust organisms and plants (Kidron et al., 2010; Kidron and Benenson, 2014). Furthermore, the higher water availability can be associated with an increase in soil nutrients such as N, K, and P (Belnap et al., 2001; Langhans et al., 2010), which is also reported in this study, together with biological factors related to nutrient fixation and cycling. For example, the observed increase in N could be due to an increase in N fixation (Belnap, 2002) and/or to increased nitrification in the soil (Delgado-Baquerizo et al., 2013), both commonly attributed to some groups of cyanobacteria.

In this sense, many studies have proposed that cyanobacteria are key elements of BSCs due to their role as primary producers and their contribution in C and N fixation (Pietrasiak et al., 2013; Bu et al., 2014; Muñoz-Martín et al., 2019). On the other hand, the increase in C and N could be related to an increase in the enzymatic activity of the soil when under the influence of BSCs (Baker and Allison, 2017). More N in the soil, for example, can be attributed to a higher urease activity, which is increased in moister soils (Delgado-Baquerizo et al., 2015; Liu et al., 2016), such as those with the presence of BSCs. The biological nitrogen mineralization process could also be positively influencing the levels of available nitrogen, which could be mediated by microbial activity, as seen in other studies (Acuña-Rodríguez et al., 2020), mainly attributed to fungi that could be also present in the BSCs. Moreover, leaf nutrient content was higher on *C. quitensis* plants exposed to BSCs compared to plants grown only in BS. These differences were more evident for N and K content, although a P, Mn, Zn, and B increase was also observed, similar to previous BSC studies (Yan, 2009; Concostrina-Zubiri et al., 2013; Havrilla et al., 2019). The high relative abundance of cyanobacteria in the BSCs could explain the increase in N in the soil and leaves. This could be related not only to the increased N fixation (as discussed previously) but also to the capacity to excrete complex polysaccharides, which can increase nitrate-reductase activity and improve root vigor in nearby plants (Flores et al., 2005; Mager and Thomas, 2011; Xu et al., 2013) thus promoting root growth and N-absorption.

The increase in soil fertility (higher organic matter and inorganic N content) has been related to an accumulation of plant biomass (DeFalco et al., 2001; Pendleton et al., 2003; Ferrenberg et al., 2018; Havrilla et al., 2020). Some studies in cold environments, such as those in western North America and northeast China, have shown that plant biomass is higher in soils with BSC cover compared with bare soils (Harper and Belnap, 2001; Zhang and Nie, 2011), similar to what we report here for *C. quitensis*. This increased biomass could be due to the positive effect of BSCs on soils, such as increased water and nutrient availability (DeFalco et al., 2001; Concostrina-Zubiri et al., 2013; Wu et al., 2013; Zhao et al., 2014; Zhang and Belnap, 2015). Moreover, BSCs’ presence could also positively influence the reproductive output (fitness) of plants (Zhang and Nie, 2011). For *C. quitensis*, we found a higher flower number when BSCs were present compared with BS. These differences, however, were not statistically significant possibly due to the small sample size. Yet, this increase in biomass investment to reproductive structures may be related to less resource-limiting conditions when BSCs are present, as stated by the resource allocation theory (Harper and Ogden, 1970).

Although in this study we have evidenced a positive effect exerted by the presence of BSCs on the soil properties and functional traits evaluated in *C. quitensis*, it is necessary to be cautious and consider new variables and other plant species inhabiting in this environment to better understand the role of BSCs on the Antarctic plant community and in dry and cold environments in general. Previous reports have described the existence of positive, neutral, and negative effects of the BSCs on plants’ performance (DeFalco et al., 2001; Eldridge and Simpson, 2002; Zhang and Nie, 2011; Godínez-Álvarez et al., 2012; Havrilla et al., 2019), where it has been observed that the responses may differ depending on the studied plant tissue, the composition of BSC, and/or the identity of the plant species.

From our results, we propose that BSCs are ecosystem engineers (*sensu* Jones et al., 1994) in Antarctic soils since they alter resource (water and nutrient) availability for the associated vascular plant species like *C. quitensis*. As such, BSCs not only change soil properties but also affect plant ecophysiology when present, possibly impacting vascular plant abundance and distribution in Antarctica. However, it is still unknown if temporal and/or spatial variations exist in the interaction between BSCs and vascular plants in Antarctica, and the exact mechanisms by which resources are transferred between these microbial communities and plants (Havrilla et al., 2019). This information could be essential to unravel the ecological role of BSCs in Antarctica and in other extreme ecosystems and to seek possible biotechnological applications of these complex soil communities.

## Conclusion

**FIGURE 5:**
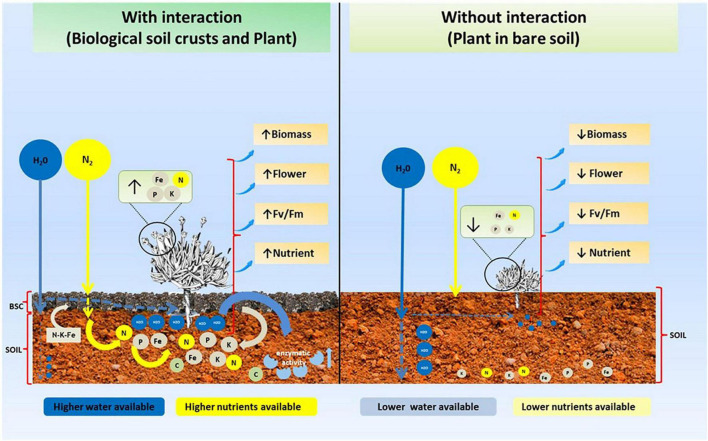
Schematic model showing the role of BSC on soil properties and their effect on vascular plants such as *C. quitensis* in Antarctica.

Biological soil crusts (BSCs) in Antarctica could be considered ecosystem engineers, directly increasing soil fertility through an increase in water and nutrient availability, which positively impact the ecophysiological performance of a native Antarctic vascular plant. This is highly relevant in Antarctica, a cold environment, where low water availability limits vascular plant growth. Thus, BSCs could have a key role in the establishment and growth of vascular plants like *C. quitensis* (Figure 5), a species that has been described as highly dependent on biotic interactions to survive the extreme conditions of Antarctica. Finally, our results suggested a bottom-up control (BSCs–vascular plant) in the Antarctic ecosystem, where the presence of BSCs increases the fitness of *C. quitensis*, which could potentially be favoring its capacity to increase its populations and distribution, a characteristic that could be valid for other vascular plants present in this environment. Thus, BSCs seem to modulate the structure of the Antarctic ecosystem, particularly plant communities.

## Data Availability Statement

The datasets presented in this study can be found in online repositories. The names of the repository/repositories and accession number(s) can be found below: PRJNA765698, https://figshare.com/articles/dataset/feature-table-16s-BSC-Antarctica_biom/16680814.

## Author Contributions

AB and MM-M designed the experiments. AB, MM-M, IA-R, and GB performed the experiments and analyzed the data. AB wrote the manuscript along with CA, MM-M, IA-R, and GB. All authors reviewed the article.

## Conflict of Interest

The authors declare that the research was conducted in the absence of any commercial or financial relationships that could be construed as a potential conflict of interest.

## Publisher’s Note

All claims expressed in this article are solely those of the authors and do not necessarily represent those of their affiliated organizations, or those of the publisher, the editors and the reviewers. Any product that may be evaluated in this article, or claim that may be made by its manufacturer, is not guaranteed or endorsed by the publisher.

## Footnotes

1
https://qiime2.org

2
https://docs.qiime2.org/2021.8/data-resources/
